# Publisher Correction: ER proteins decipher the tubulin code to regulate organelle distribution

**DOI:** 10.1038/s41586-022-04656-7

**Published:** 2022-03-23

**Authors:** Pengli Zheng, Christopher J. Obara, Ewa Szczesna, Jonathon Nixon-Abell, Kishore K. Mahalingan, Antonina Roll-Mecak, Jennifer Lippincott-Schwartz, Craig Blackstone

**Affiliations:** 1 Cell Biology Section, Neurogenetics Branch, National Institute of Neurological Disorders and Stroke, National Institutes of Health, Bethesda, MD USA; 2 Janelia Research Campus, Howard Hughes Medical Institute, Ashburn, VA USA; 3 Cell Biology and Biophysics Section, National Institute of Neurological Disorders and Stroke, National Institutes of Health, Bethesda, MD USA; 4 Biochemistry and Biophysics Center, National Heart, Lung and Blood Institute, National Institutes of Health, Bethesda, MD USA; 5 MassGeneral Institute for Neurodegenerative Disease, Massachusetts General Hospital, Charlestown, MA USA; 6 Department of Neurology, Massachusetts General Hospital and Harvard Medical School, Boston, MA USA; 7 Present Address: Cambridge Institute for Medical Research, Cambridge, UK

**Keywords:** Microtubules, Endoplasmic reticulum

Correction to: *Nature* 10.1038/s41586-021-04204-9 Published online 15 December 2021

In the Methods section of this Article, in the ‘CRISPR–Cas9 gene editing’ section, the sequences used as a homologous recombination template for the two proteins (CLIMP63 and calreticulin) should have been separated. The original Article has been corrected online

