# Supplementary material for: ER proteins decipher the tubulin code to regulate organelle distribution
Source: Nature. 2021 Dec 15;601(7891):132–8. doi: 10.1038/s41586-021-04204-9 (PMC8732269; doi:10.1038/s41586-021-04204-9)

nature portfolio

https://doi.org/10.1038/s41586-021-04204-9

Supplementary information

ER proteins decipher the tubulin code to regulate organelle distribution

In the format provided by the authors and unedited

SI Guide

ER Proteins Decipher the Tubulin Code to Regulate Organelle Distribution

Pengli Zheng, Christopher J. Obara, Ewa Szczesna, Jonathon Nixon-Abell, Kishore K. Mahalingan, Antonina Roll-Mecak, Jennifer Lippincott-Schwartz, Craig Blackstone

Table of contents

Supplementary text

SI Figure 1, Uncropped western blotting data from Figure 3 and Extended Data (ED) Figure

1. SI Figure 2, Uncropped western blotting data from ED Figure 3.

SI Figure 3, Uncropped western blotting data from ED Figure 3.

SI Figure 4, Uncropped western blotting data from ED Figures 4,5,6.

SI Figure 5, Uncropped western blotting data from ED Figures 6,7,8.

SI Figure 6, Uncropped western blotting data from ED Figure 10.

SI Figure 7, Uncropped western blotting data from ED Figure 10.

Proteins were detected by western blotting using the indicated antibodies, and the red rectangles show the cropping location. GAPDH or α-tubulin serve as sample processing controls that were run on separate gels.

Supplementary text

Reasoning for spatial density estimation and asymmetry calculation

In diffraction-limited imaging, fluorescence emission from a sample is generally quantitative. Consequently, we assume the fluorescence in each pixel is proportional to the amount of protein (or organelle) present within the focal volume from which the pixel was derived. Thus, the ratio of the intensity of a single pixel to the sum of all the pixels in a particular cell represents the probability that an arbitrarily selected piece of the labeled object is localized within that pixel. In other words, it serves as a pixel-based probability mass function, which estimates the true probability density function (pdf) at the resolution of the microscope. The estimated value of the pdf in a pixel at coordinates (x, y) is given by $\rho_{x,y}$ such that:

$$\rho_{x,y} = \frac{I_{x,y}}{\sum_{cell} I} \qquad \text{and} \qquad \iint_{cell} \rho_{x,y}\, dx\, dy \equiv 1$$

where $I_{x,y}$ is the intensity of the pixel. Because it is derived from a ratio, this distribution is independent of expression level or staining efficiency and only assumes that all molecules are equally likely to be stained/visualized. From simple probability theory, the expected value of an observable k can be calculated by integrating over the organelle or any desired sub-region:

$$\langle k \rangle = \iint k\, \rho_{x,y}\, dx\, dy$$

In the applications used in this paper, this is practically accomplished by estimating the value from the corresponding sum over the pixels in the region. In theory, this is applicable to any spatially defined variable, but for the purposes of this paper we are primarily interested in the relative distribution of the organelles through the cytoplasm. We utilized polar coordinates in the 2D projection with the origin centered in the nucleus, so the radial coordinate alone could be used to easily quantify the tendency of an organelle to stay close to the nucleus or extend into the periphery. (When integrated over θ, the polar form of the pdf represents the probability of an arbitrary bit of fluorescence falling in a series of very thin concentric rings spreading away from

the center of the cell, see Extended Data Fig. 2). The average distance of the organelle from the center of the cell is then given by:

$$\langle r \rangle = \iint_{0,0}^{R,2\pi} r\rho_{x,y}\, dr\, d\theta$$

This proves to be a robust mechanism for quantifying the distribution of structures with very complex morphologies such as ER and microtubules.

Since some knock-out cell lines showed highly asymmetric distributions of ER, we reasoned that a similar approach could be utilized to quantify the degree of asymmetry. To avoid weighting the result by the radius (this would unfairly favor structural organization in the periphery over the perinuclear region), we calculated a "coefficient of asymmetry" by performing the integral across the full diameter of the cell, using a sign function sgn(r) as the mathematical operator to make one side of the cell arbitrarily positive and the other negative. We then took the absolute value of each diameter to quantify the net "inequality" in total fluorescence between the two sides:

$$\langle a \rangle = \int_{0}^{\pi} \left| \int_{-R}^{R} sgn(r) r\rho_{x,y}\, dr \right| d\theta$$

This ensures that fluorescence on one side or the other of the cell only contributes to the coefficient of asymmetry if there is not a corresponding amount of fluorescence on the other side, with a total integral equal to zero if the cell is completely uniform and equal to 1 if it is completely distributed asymmetrically.

Zheng et al., SI Figure 1

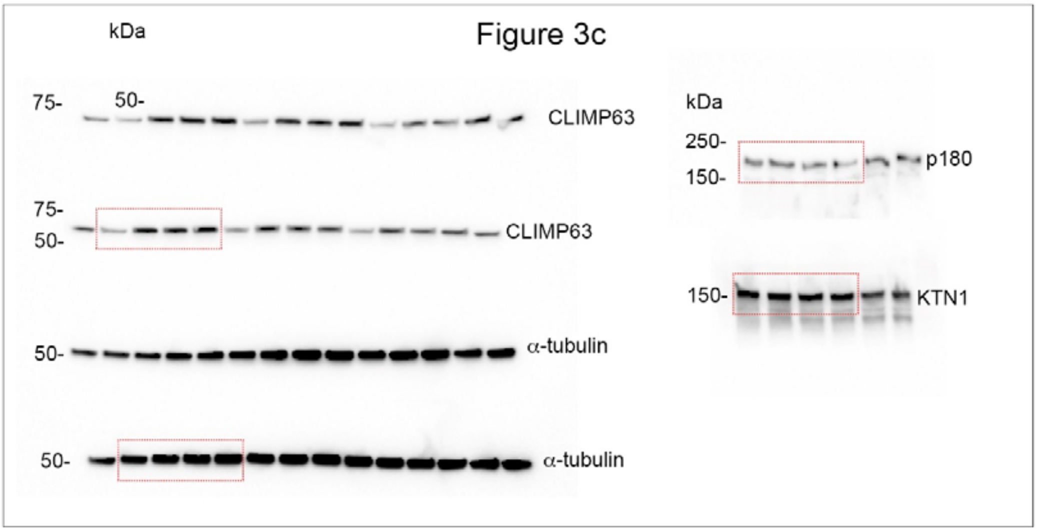

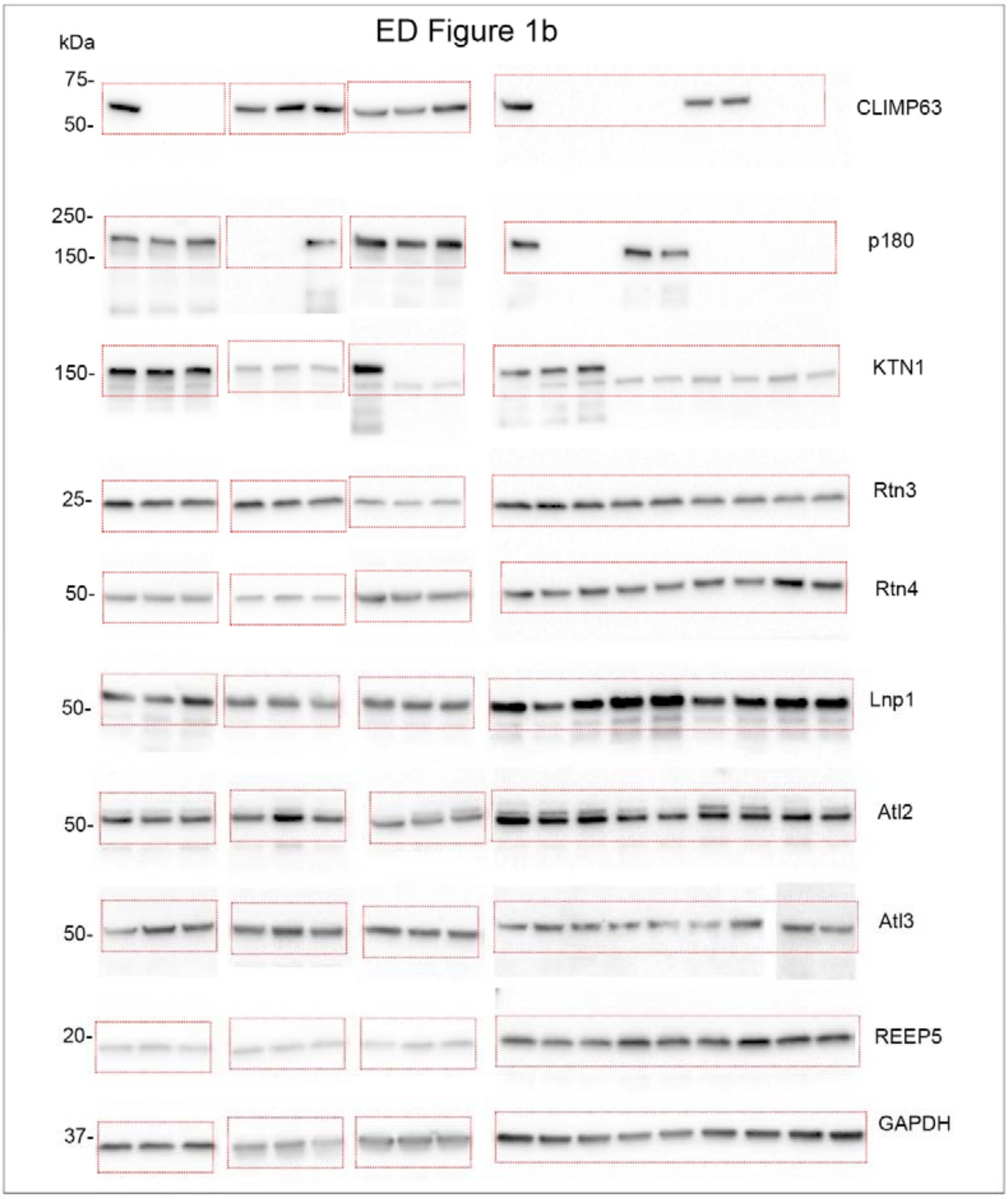

Zheng et al., SI Figure 2

ED Figure 3a

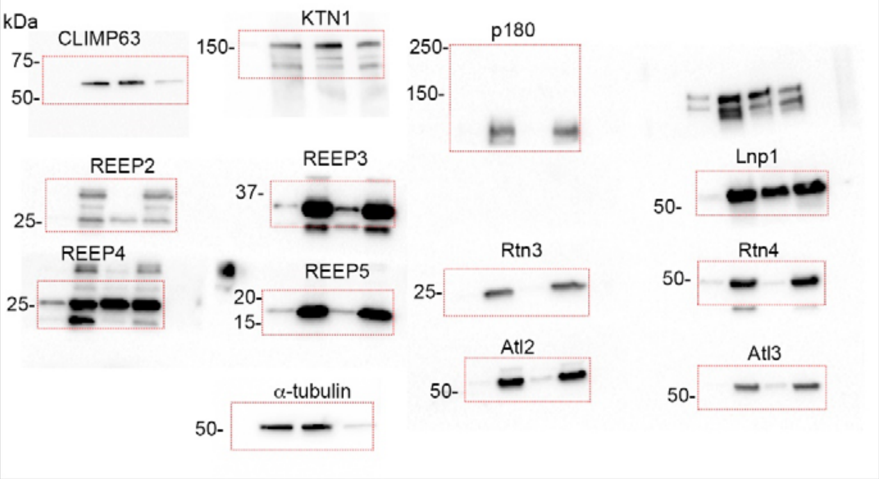

ED Figure 3b

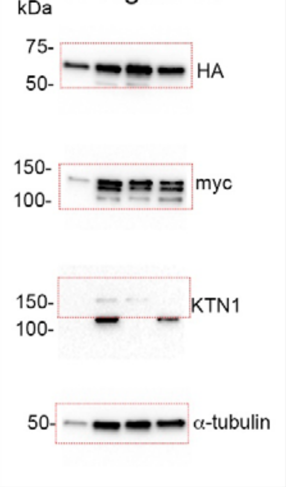

ED Figure 3c

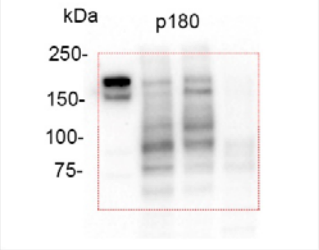

ED Figure 3d

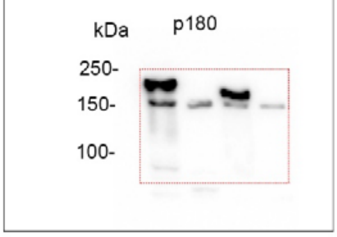

ED Figure 3e

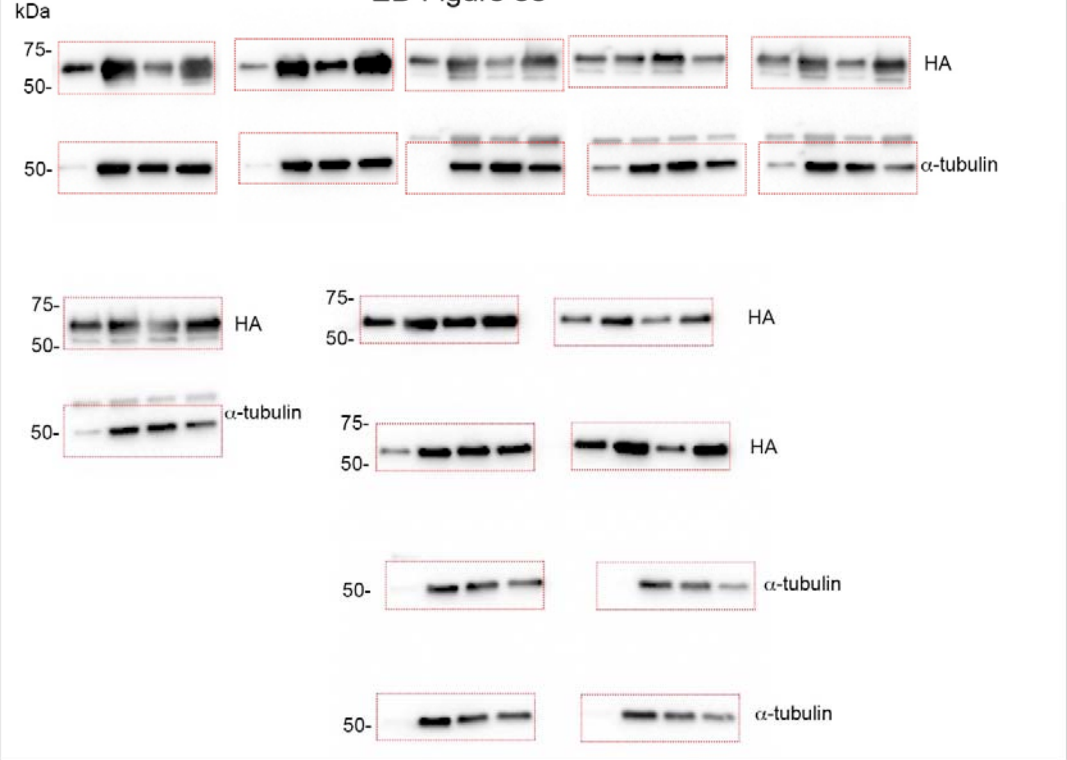

Zheng et al., SI Figure 3

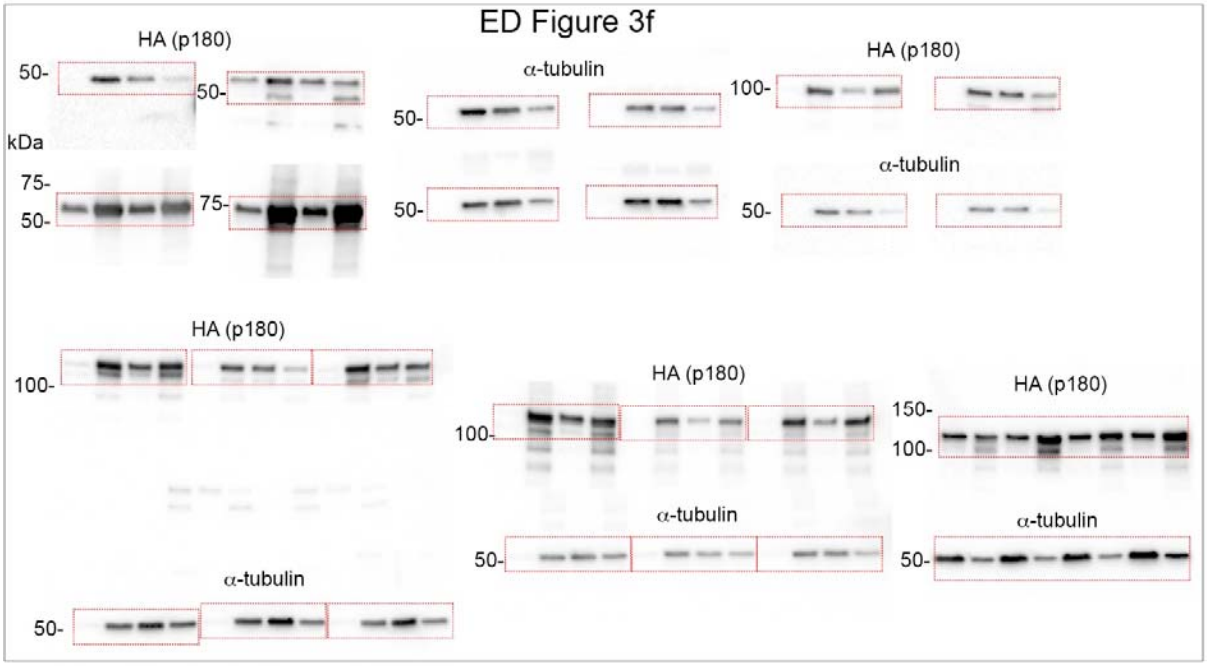

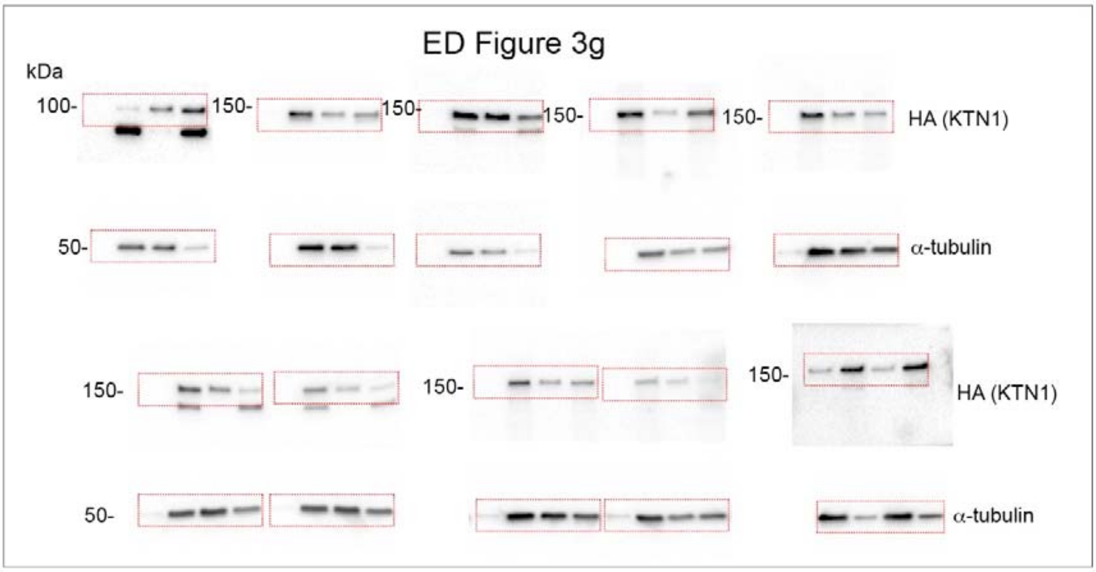

Zheng et al., SI Figure 4

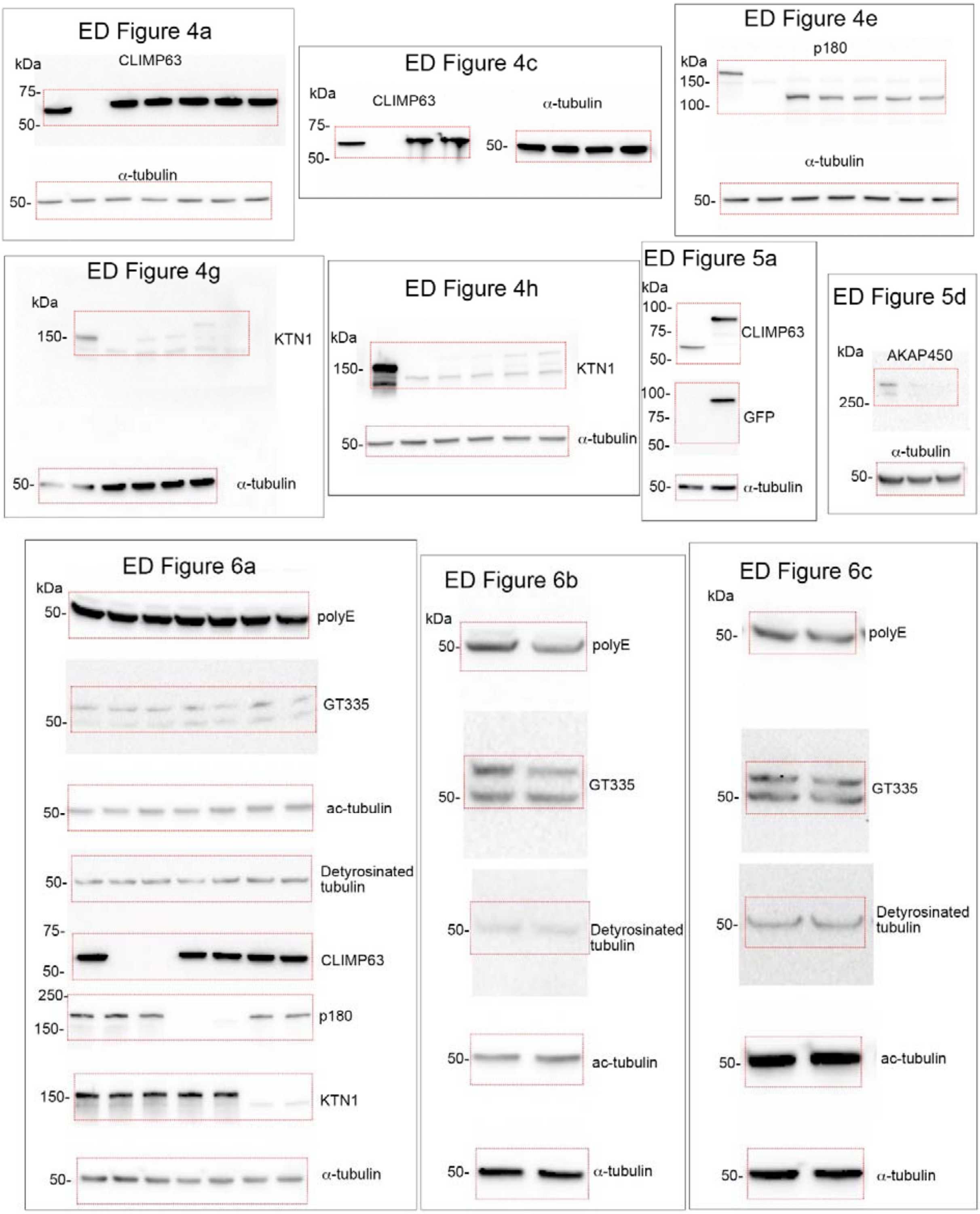

Zheng et al., SI Figure 5

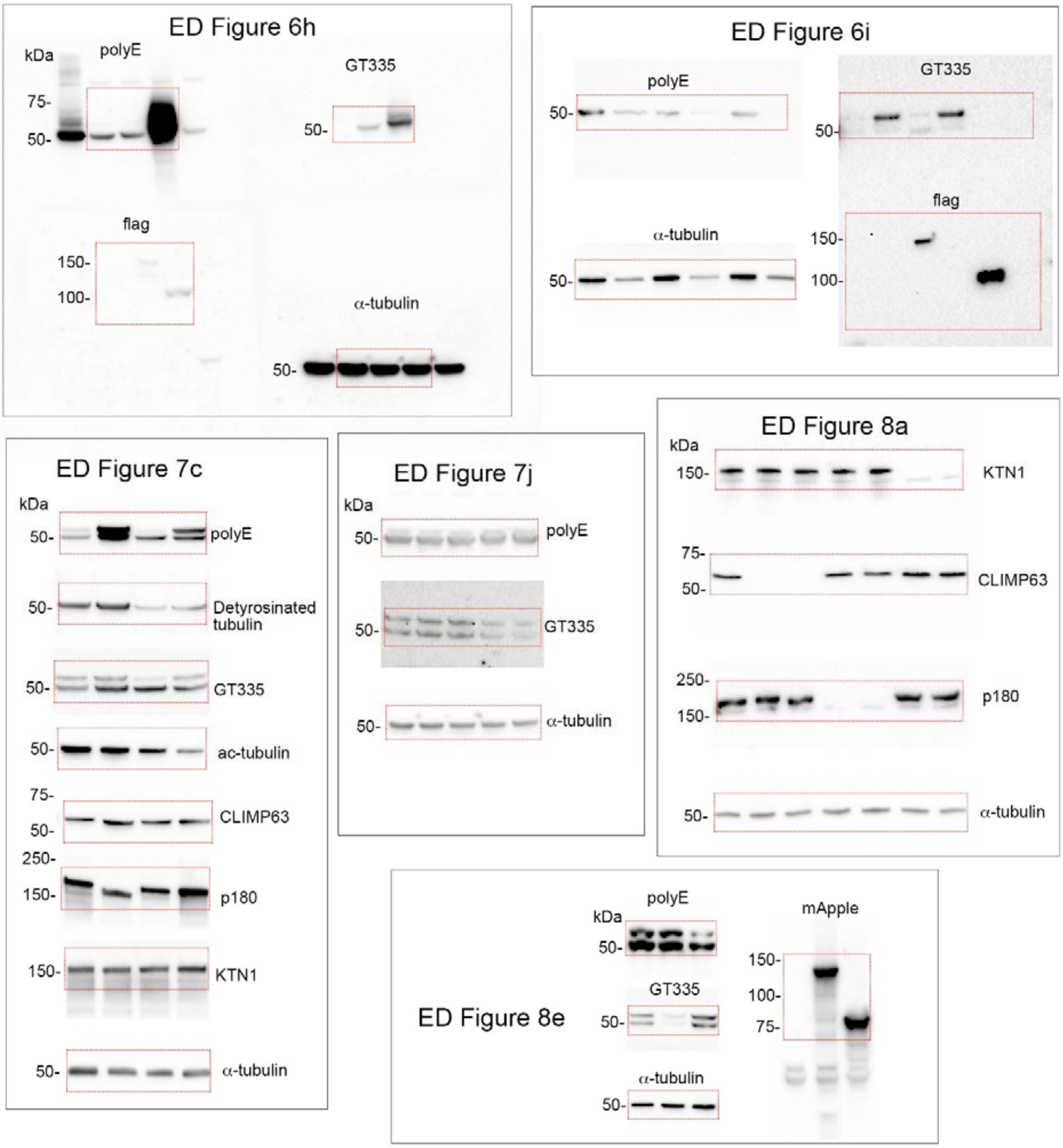

Zheng et al., SI Figure 6

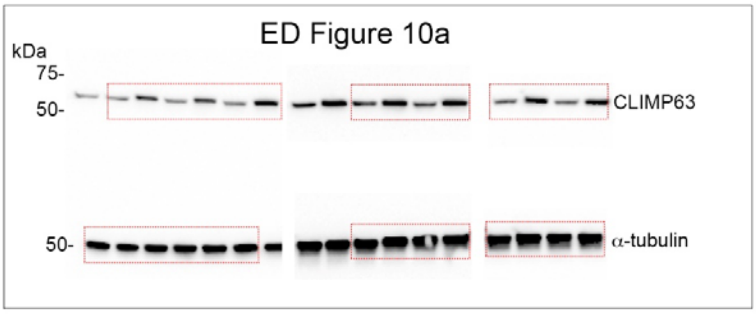

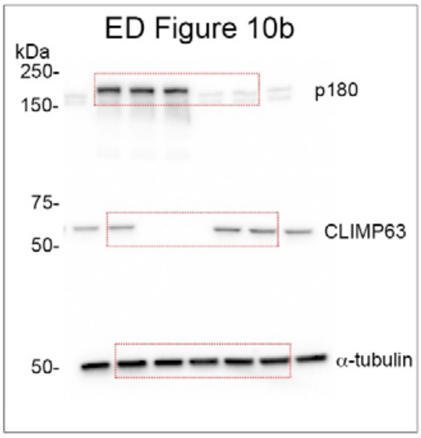

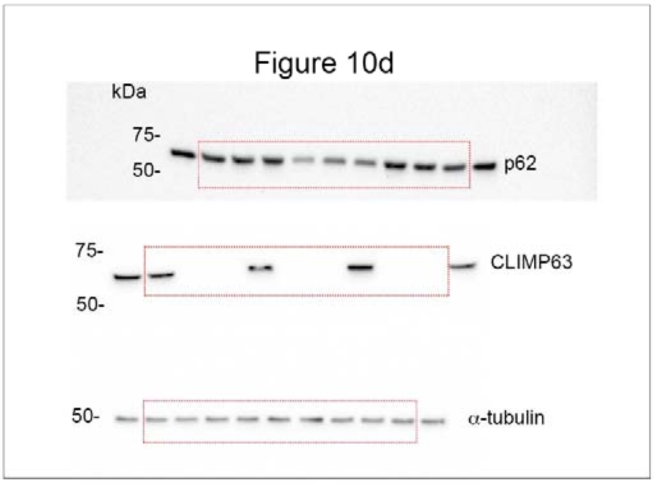

Zheng et al., SI Figure 7

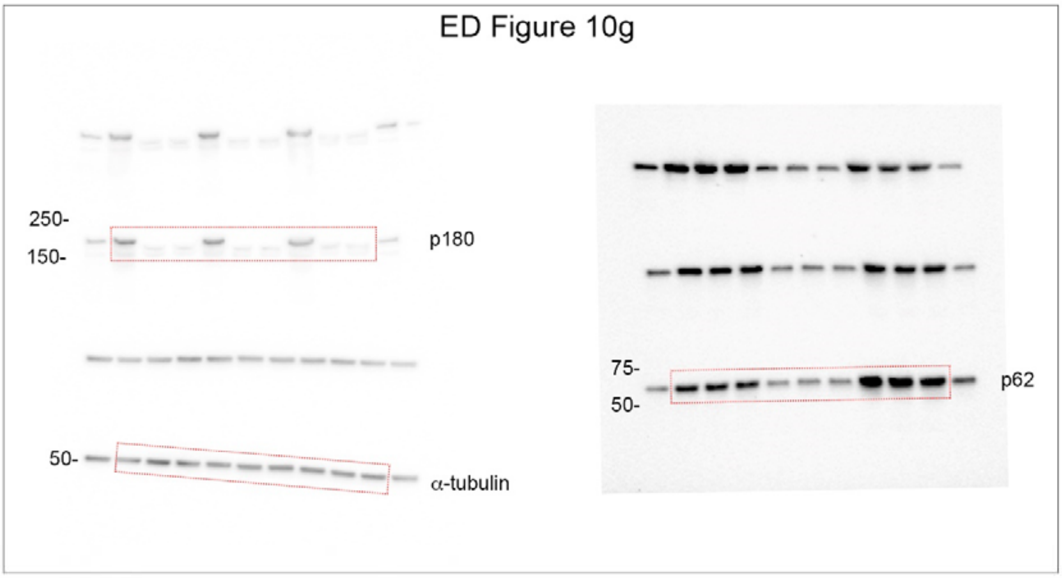

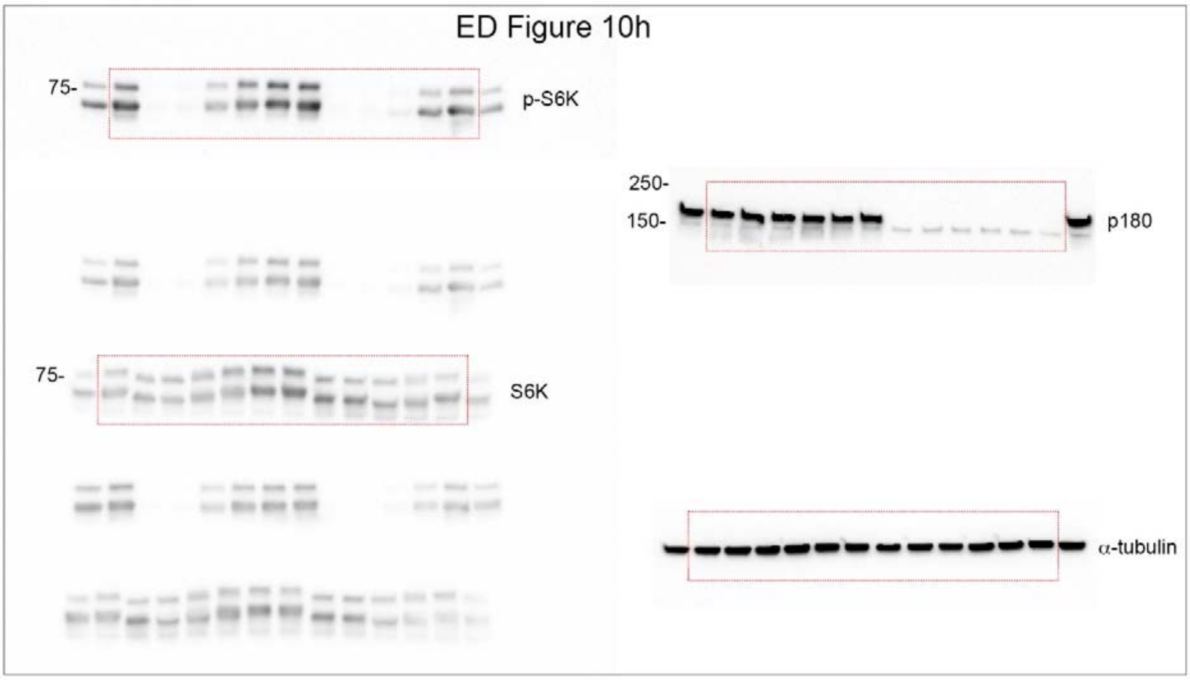

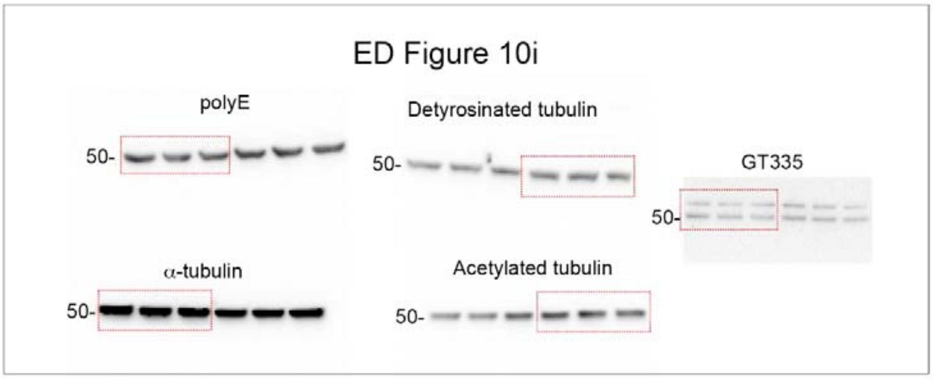

Supplement: Supplementary file 1 — This file contains Supplementary Text and Supplementary Figs 1–7. [file 41586_2021_4204_MOESM1_ESM.pdf]
