# Supplementary material for: ER proteins decipher the tubulin code to regulate organelle distribution
Source: Nature. 2021 Dec 15;601(7891):132–8. doi: 10.1038/s41586-021-04204-9 (PMC8732269; doi:10.1038/s41586-021-04204-9)
Supplement: Supplementary file 2 — Reporting Summary [file 41586_2021_4204_MOESM2_ESM.pdf]

nature portfolio

Corresponding author(s): Craig Blackstone

Last updated by author(s): Oct 7, 2021

Reporting Summary

Nature Portfolio wishes to improve the reproducibility of the work that we publish. This form provides structure for consistency and transparency in reporting. For further information on Nature Portfolio policies, see our Editorial Policies and the Editorial Policy Checklist.

Statistics

For all statistical analyses, confirm that the following items are present in the figure legend, table legend, main text, or Methods section.

n/a	Confirmed	
☐	☒	The exact sample size (n) for each experimental group/condition, given as a discrete number and unit of measurement
☐	☒	A statement on whether measurements were taken from distinct samples or whether the same sample was measured repeatedly
☐	☒	The statistical test(s) used AND whether they are one- or two-sided<br>*Only common tests should be described solely by name; describe more complex techniques in the Methods section.*
☐	☒	A description of all covariates tested
☐	☒	A description of any assumptions or corrections, such as tests of normality and adjustment for multiple comparisons
☐	☒	A full description of the statistical parameters including central tendency (e.g. means) or other basic estimates (e.g. regression coefficient) AND variation (e.g. standard deviation) or associated estimates of uncertainty (e.g. confidence intervals)
☐	☒	For null hypothesis testing, the test statistic (e.g. F, t, r) with confidence intervals, effect sizes, degrees of freedom and P value noted<br>*Give P values as exact values whenever suitable.*
☒	☐	For Bayesian analysis, information on the choice of priors and Markov chain Monte Carlo settings
☐	☒	For hierarchical and complex designs, identification of the appropriate level for tests and full reporting of outcomes
☒	☐	Estimates of effect sizes (e.g. Cohen's d, Pearson's r), indicating how they were calculated

Our web collection on statistics for biologists contains articles on many of the points above.

Software and code

Policy information about availability of computer code

Data collection	No software was used to collect the data
Data analysis	Computer algorithms can be accessed at https://github.com/cjobara/ProbabilityDensityIntegrator.

For manuscripts utilizing custom algorithms or software that are central to the research but not yet described in published literature, software must be made available to editors and reviewers. We strongly encourage code deposition in a community repository (e.g. GitHub). See the Nature Portfolio guidelines for submitting code & software for further information.

Data

Policy information about availability of data

All manuscripts must include a data availability statement. This statement should provide the following information, where applicable:

- Accession codes, unique identifiers, or web links for publicly available datasets
- A description of any restrictions on data availability
- For clinical datasets or third party data, please ensure that the statement adheres to our policy

All data are presented in the main text or Supplementary Materials.

Field-specific reporting

Please select the one below that is the best fit for your research. If you are not sure, read the appropriate sections before making your selection.

☒ Life sciences ☐ Behavioural & social sciences ☐ Ecological, evolutionary & environmental sciences

For a reference copy of the document with all sections, see nature.com/documents/nr-reporting-summary-flat.pdf

Life sciences study design

All studies must disclose on these points even when the disclosure is negative.

Sample size	No statistical method was used to predetermine sample size.
Data exclusions	No data were excluded
Replication	At least three repeats were performed unless otherwise stated
Randomization	All groups were randomly assigned and every group represents a distinct treatment or condition.
Blinding	Data were not analyzed in a double-blinded manner. The phenotypes of different groups are obvious and double-blindness can't be applied.

Reporting for specific materials, systems and methods

We require information from authors about some types of materials, experimental systems and methods used in many studies. Here, indicate whether each material, system or method listed is relevant to your study. If you are not sure if a list item applies to your research, read the appropriate section before selecting a response.

Materials & experimental systems

n/a	Involved in the study	
☐	☒	Antibodies
☐	☒	Eukaryotic cell lines
☒	☐	Palaeontology and archaeology
☒	☐	Animals and other organisms
☒	☐	Human research participants
☒	☐	Clinical data
☒	☐	Dual use research of concern

Methods

n/a	Involved in the study	
☒	☐	ChIP-seq
☒	☐	Flow cytometry
☒	☐	MRI-based neuroimaging

Antibodies

Antibodies used	mouse monoclonal anti-AKAP450 (BD Biosciences, 611518, Clone 7/AKAP450, immunoblot 1:250), rabbit polyclonal anti-Atlastin2 (Bethyl Laboratories, A303-333A, immunoblot 1:500), rabbit polyclonal anti-Atlastin3 (Proteintech, 16921-1-AP, immunoblot 1:1000), rabbit monoclonal anti-Catalase (Cell Signaling Technology, 12980, clone D4P7B, immunofluorescence 1:800), mouse monoclonal anti-Climp63 (Enzo, ALX-804-604, clone G1/296, immunofluorescence 1:500 immunoblot 1:5000), mouse monoclonal anti-Flag M2 (Sigma-Aldrich, F1804, clone M2, immunoblot 1:1000), rabbit polyclonal anti-GFP (MBL, 598, immunoblot 1:5000, immunofluorescence 1:500), mouse monoclonal anti-GM130 (BD Biosciences, 610822, Clone 35/GM130, immunofluorescence 1:200), rabbit polyclonal anti-GM130 (Proteintech, 11308-1-AP, immunofluorescence 1:200), mouse monoclonal anti-HA (Covance, MMS-101P, clone 16B12, immunofluorescence 1:500, immunoblot 1:5000), rabbit polyclonal anti-kinectin (Proteintech, 19841, immunoblot 1:2000), rabbit monoclonal anti-kinectin (Cell Signaling Technology, 13243, clone D5F7J, immunofluorescence 1:100), mouse monoclonal anti-Lamp1 (DSHB, clone 1D4B, immunofluorescence 1:2000), rabbit polyclonal anti-LC3 (Cell Signaling Technology, 4108, immunofluorescence 1:200, immunoblot 1:1000), rabbit polyclonal anti-Lunapark (Sigma-Aldrich, HPA014205, immunoblot 1:250), mouse monoclonal anti-Myc (Santa Cruz, sc-40, clone 9E10, immunoblot 1:2000), rabbit polyclonal anti-p180 (Thermo Fisher Scientific, PA5-21392, immunofluorescence 1:500, immunoblot 1:5000), rabbit polyclonal anti-Pericentrin (Abcam, ab4448, immunofluorescence 1:1000), rabbit polyclonal anti-polyglutamation (polyE) (AdipoGen, AG-25B-0030, immunofluorescence 1:200, immunoblot 1:1000), mouse monoclonal anti-glutamylation clone GT335 (AdipoGen, AG-20B-0020, immunofluorescence 1:200, immunoblot 1:200), rabbit polyclonal anti-REEP2 (Proteintech, 15684, immunoblot 1:3000), rabbit polyclonal anti-REEP3 (Abcam, ab106463, immunoblot 1:1000), rabbit polyclonal anti-REEP4 (Proteintech, 26650, immunoblot 1:1000), rabbit polyclonal anti-REEP5 (Proteintech, 14643, immmunoblot 1:1000), rabbit polyclonal anti-reticulon3 (Proteintech, 12055, immunoblot 1:2000), rabbit polyclonal anti-reticulon4 (Proteintech, 10740, immunoblot 1:1000), rabbit polyclonal anti-RPL3 (Proteintech, 66130, immunofluorescence 1:100), rabbit polyclonal anti-TOM20 (Santa Cruz, sc-11415, immunofluorescence 1:1000), mouse monoclonal anti-TOM20 (BD Biosciences, 612278, Clone 29/Tom20, immunofluorescence 1:1000), rabbit polyclonal anti-TRAPα (Proteintech, 10583, immunofluorescence 1:50), rat monoclonal anti-α-tubulin Alexa Fluor 647 (Abcam, ab195884, clone YOL1/34, immunofluorescence 1:50), mouse monoclonal anti-α-tubulin (Proteintech, 66031, clone 1E4C11, immunofluorescence 1:1000, WB 1:10000), mouse monoclonal anti-β-tubulin (Proteintech, 66240, clone 1D4A4, immunofluorescence 1:1000). Alexa Fluor

	405/488/568/633 conjugated goat anti-rabbit/mouse IgG (H+L) highly cross-adsorbed secondary antibodies were from Thermo Fisher Scientific. HRP-conjugated goat anti-mouse/rabbit secondary antibodies were from Santa Cruz Biotechnology.
Validation	ALL antibodies were commercial and verified by the company with the following applications: mouse monoclonal anti-AKAP450 (WB), rabbit polyclonal anti-Atlastin2 (WB, IP), rabbit polyclonal anti-Atlastin3 (WB, IP), rabbit monoclonal anti-Catalase (WB, IP, IF), mouse monoclonal anti-Climp63 (WB, IP, IF), mouse monoclonal anti-Flag M2 (WB, IP, IF), rabbit polyclonal anti-GFP (WB, IP, IF), mouse monoclonal anti-GM130 (WB, IF), rabbit polyclonal anti-GM130 (WB, IF), mouse monoclonal anti-HA (WB, IP, IF), rabbit polyclonal anti-kinectin (WB, IP, IF), rabbit monoclonal anti-kinectin (WB, IP, IF), mouse monoclonal anti-Lamp1 (IF), rabbit polyclonal anti-LC3 (WB, IF), rabbit polyclonal anti-Lunapark (WB), mouse monoclonal anti-Myc (WB, IP), rabbit polyclonal anti-p180 (WB, IF), rabbit polyclonal anti-Pericentrin (IF), rabbit polyclonal anti-polyglutamation (polyE) (WB, IF), mouse monoclonal anti-glutamylation clone GT335 (WB, IF), rabbit polyclonal anti-REEP2 (WB), rabbit polyclonal anti-REEP3 (WB), rabbit polyclonal anti-REEP4 (WB), rabbit polyclonal anti-REEP5 (WB), rabbit polyclonal anti-reticulon3 (WB), rabbit polyclonal anti-reticulon4 (WB), rabbit polyclonal anti-RPL3 (WB, IF), rabbit polyclonal anti-TOM20 (WB, IF), mouse monoclonal anti-TOM20 (WB, IF), rabbit polyclonal anti-TRAPα (WB, IF), rat monoclonal anti-α-tubulin Alexa Fluor 647 (IF), mouse monoclonal anti-α-tubulin (WB, IF), mouse monoclonal anti-β-tubulin (WB, IF).

Eukaryotic cell lines

Policy information about cell lines

Cell line source(s)	All cell lines were obtained from the American Type Culture Collection (ATCC) including HEK293T (CRL-11268), COS7 (CRL-1651), HeLa (CCL-2), RPE1 (CRL-4000) and U2OS (HTB-96) cells
Authentication	All cell lines were authenticated by ATCC using STR profiling.
Mycoplasma contamination	All cell lines tested negative for mycoplasma contamination.
Commonly misidentified lines (See ICLAC register)	no commonly misidentified cell lines were used in the study.
